# DVL1 and DVL3 require nuclear localisation to regulate proliferation in human myoblasts

**DOI:** 10.1038/s41598-022-10536-x

**Published:** 2022-05-19

**Authors:** Johanna Pruller, Nicolas Figeac, Peter S. Zammit

**Affiliations:** King’s College London, Randall Centre for Cell and Molecular Biophysics, London, SE1 1UL UK

**Keywords:** Cell division, Cell signalling

## Abstract

WNT signalling is essential for regulating a diverse range of cellular processes. In skeletal muscle, the WNT pathway plays crucial roles in maintenance of the stem cell pool and myogenic differentiation. Focus is usually directed at examining the function of central components of the WNT pathway, including β-CATENIN and the GSK3β complex and TCF/LEF transcription factors, in tissue homeostasis and cancer. Other core components of the WNT pathway though, are three dishevelled (DVL) proteins: membrane associated proteins that propagate WNT signalling from membrane to nucleus. Here we examined DVL function in human myogenesis and the muscle-related cancer alveolar rhabdomyosarcoma. We demonstrate that DVL1 and DVL3 are necessary for efficient proliferation in human myoblasts and are important for timely myogenic differentiation. DVL1 and DVL3 also contribute to regulation of proliferation in rhabdomyosarcoma. DVL1 or DVL3 must be present in the nucleus to regulate proliferation, but they operate through different protein domains: DVL3 requires the DIX and PDZ domains, while DVL1 does not. Importantly, DVL1 and DVL3 activity is independent of markedly increased translocation of β-CATENIN to the nucleus, normally a hallmark of active canonical WNT signalling.

## Introduction

Skeletal muscle is a highly organised tissue, accounting for approximately 38% of body mass in men and 31% in women^1^. The force-generating unit of skeletal muscle is the muscle fibre, a syncytial cell formed by the fusion of myogenic precursor cells. During embryonic development, WNT signalling is needed for successful somitogenesis^2^ and subsequent establishment of the dermomyotome^3^, which contains the precursor cells for most skeletal musculature. While the role of WNT signalling during embryonic development is well characterised, its function during muscle regeneration is less known. Satellite cells are the resident stem cells of postnatal skeletal muscle, responsible for providing myoblast progeny for growth, homeostasis and repair^4^ and their dysfunction can contribute to muscle disease^5^. Freshly isolated myofibres and satellite cells express low levels of WNT ligands, but after injury, many WNT ligands (WNT3a, 5a, 5b, 7a, 7b) are expressed and secreted^6^. WNT signalling contributes to regulation of satellite cell proliferation, differentiation and self-renewal^7^, and must be suppressed for regeneration to complete in mouse^8^. WNT signalling is also differently regulated in various muscular diseases, such as in facioscapulohumeral muscular dystrophy^9,10^. However, how WNT signalling is regulated during muscle regeneration at the molecular level is not well understood.

WNT signalling pathways are commonly divided into: (i) Canonical WNT signalling, relying on translocation of β-CATENIN into the nucleus and interaction with transcription factors including TCF/LEF to control gene expression, (ii) Non-canonical planar cell polarity (PCP) WNT signalling which regulates cytoskeletal rearrangements via Rac/Rho dependent activation of JNK signalling or cell adhesion and movement via Ca^2+^ and NFAT signalling^11,12^. Early mediators in each WNT pathway are the dishevelled proteins (DVL), which facilitate inhibition of the destruction complex targeting β-CATENIN at the cell membrane.

The three human isoforms, DVL1, DVL2 and DVL3, share more than 90% amino acid sequence homology, especially in their highly conserved DIX, PDZ and DEP domains^13^. During canonical WNT signalling, DVL proteins are recruited to the cell membrane. This prevents GSK3β from phosphorylating β-CATENIN, allowing non-phosphorylated (active) β-CATENIN to translocate to the nucleus^14^. However, DVL proteins also contain nuclear localisation (NLS) and nuclear export (NES) signals, and the ability of DVLs to shuttle between cytoplasm and nucleus is essential for effective canonical WNT signalling^15^. Translocation of DVL proteins into the nucleus is regulated by FOXK transcription factors^16^. During non-canonical WNT signalling, DVL complexing with Daam1 activates Rho and associated kinases, which influence cell motility^17^.

DVL1, DVL2 and DVL3 are expressed during development, and have both unique and redundant functions. Defective canonical WNT signalling in *Dvl1*^-/-^ mice impairs central nervous system function, culminating in reduced social abilities^18^, while *Dvl2*^-/-^ mice display defective cardiac morphogenesis and somite segmentation^19^. *Dvl3*^-/-^ mice are embryonic lethal, with *Dvl3*^*-/-*^* ; Dvl1*^*-/-*^ showing earlier lethality at E13.5-E15.5 and *Dvl3*^*-/-*^* ; Dvl2*^+*/-*^ dying at E9.5^20^. All three DVLs, but especially DVL2, are involved in neural tube closure^21^. Deletion of all *Dvl* alleles decreases canonical WNT dependent mesoderm gene expression and mesoderm formation but low levels of *Dvl* expression from a single allele can be sufficient to at least rescue early development^22,23^.

DVLs not only facilitate WNT signalling by preventing destruction of β-CATENIN to allow it to translocation to the nucleus^24^, but in skeletal myogenesis, DVLs also directly control establishment of Acetylcholine Receptor clustering^25^. Most data about DVL function in skeletal muscle are centred around DVL2, as it is the most abundant isoform^26^. Stabilisation of Dvl2 and protection from autophagy increases canonical WNT signalling and so proliferation and differentiation in murine C2C12 myoblasts and protects them against atrophy^24,27^. Increased DVL stability generally precedes increased WNT signalling, as more DVL at the cell membrane facilitates DVL oligomerisation and signal propagation^28^. This is shown in rats, where exercise increases the association of DVL with GSK3β, which is accompanied by increased β-CATENIN dephosphorylation^29^. Emerging evidence indicates that localisation of DVL in the nucleus is crucial for proper canonical WNT signalling^16^. Additionally, nuclear-located DVL represses NFκB signalling^30^, and mutation of the NES sequence in DVL inhibits cytosolic translocation of phosphorylated YAP^31^, which influences satellite cell fate^32^.

The conserved DIX, PDZ and DEP domains of DVLs are implicated in different ways to regulate the WNT pathway. The DEP domain is predominantly involved in recruitment of DVL to the membrane. DEP and DIX domains are necessary for DVL homo- and heterodimerization and subsequent activation of canonical WNT signalling (reviewed in^22^). The PDZ domain is necessary for physical interaction between DVL and various WNT signalling agonists or antagonists^33,34^, among them Frizzled (FZD) receptors^35,36^, and it functions in both canonical and PCP pathways^17^.

The WNT pathway is also deregulated in cancer, where hyperactive WNT signalling facilitates invasion and metastasis, and therefore provides a potential therapeutic target^37^. Of particular relevance here, the childhood onset soft tissue cancer rhabdomyosarcoma is characterised by increased WNT signalling and also expresses many genes associated with the skeletal muscle lineage^38^. In particular, DVL proteins play a role in regulating proliferation in many cancer cell types^39,40^.

Most studies consider the DVL proteins only as a component of WNT signalling propagation, and tend not to directly test the functional consequence of DVL manipulation. Given the role of WNT signalling in regenerative myogenesis and the ability of DVL2 to enhance proliferation, we were interested in elucidating the roles of DVL1-3 in regulation of myoblast proliferation and differentiation.

Here, we investigate the role of DVL proteins in human skeletal myogenesis. We found that DVL1 and DVL3 are essential to maintain proliferation in human myoblasts in a β-CATENIN independent way, and that nuclear localisation of DVL1 or DVL3 is crucial for their function. In addition, DVL1/3 are also required for timely entry into myogenic differentiation. However, DVL1 and DVL3 regulate proliferation via different mechanisms, since DVL3 requires the presence of both the DIX and PDZ domains, while DVL1 is unaffected by individual deletion of these domains. Importantly, DVL1 and DVL3 also regulate proliferation in alveolar rhabdomyosarcoma (ARMS) cells.

## Results

### Expression profile of DVL isoforms during human skeletal myogenesis

**Figure 1:**
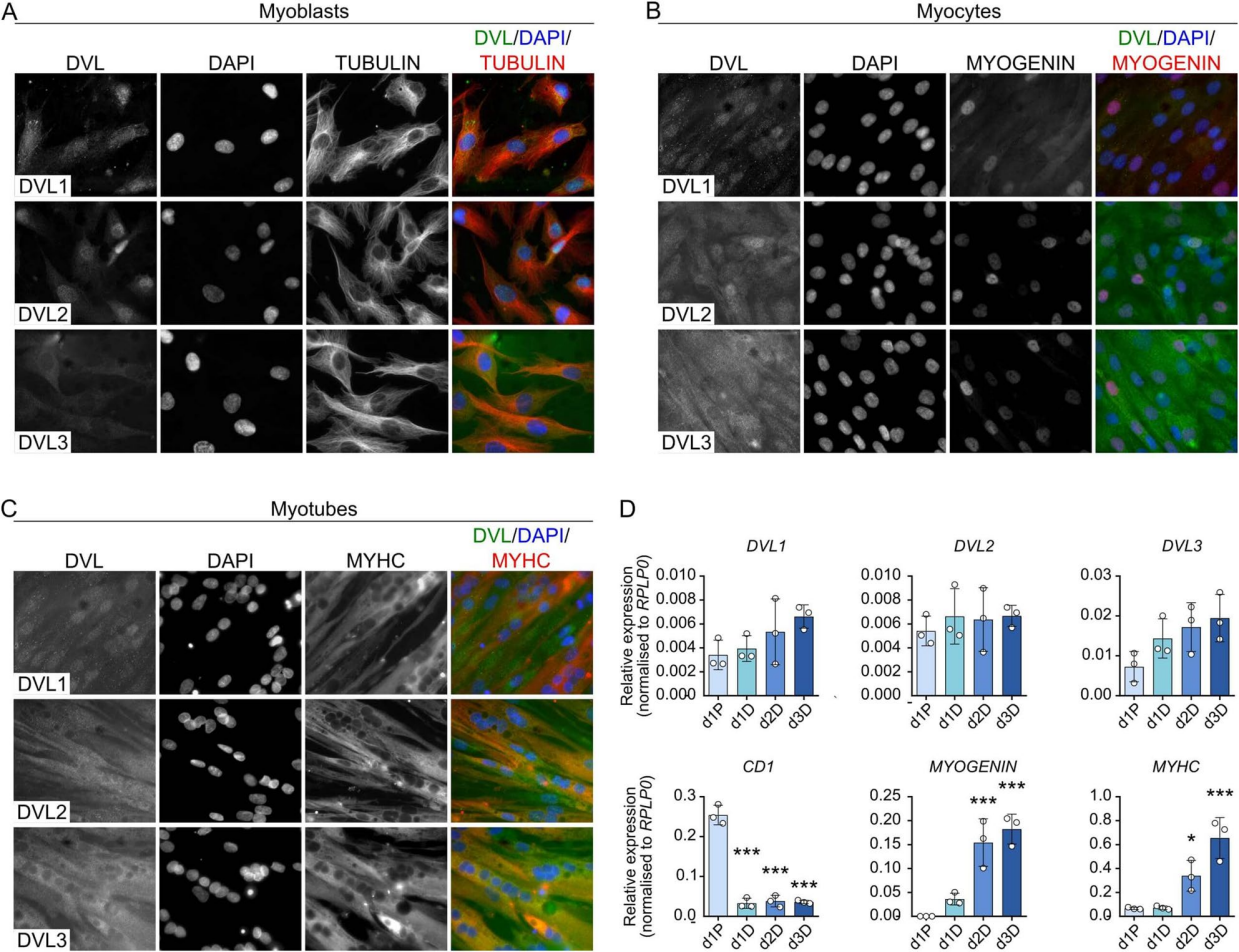
Localisation and expression profile of DVL1-3 during human skeletal myogenesis. (**A**) Localisation of DVL1-3 in proliferating human C25 myoblasts. (**B**) Localisation of DVL isoforms in C25 myoblasts after two days of differentiation and (**C**) localisation of DVL1-3 isoforms in multinucleated C25 myotubes after three days of differentiation. (**D**) Gene expression profile for *DVL1-3,* proliferation marker *CD1* and early *(MYOGENIN)* and late *(MyHC: MYH3, MYH8* and *MYH2)* differentiation markers in C25 myoblasts on day (d) 1 in proliferation (d1P) and on days (d) 1, 2 and 3 of differentiation (d1D, d2D and d3D). Data is represented as mean ± SD. *N* = 3, statistically significant differences were assessed using a One-WAY ANOVA with Dunett’s post hoc test, comparing each time-point to proliferation where an asterisk denotes *p* < 0.05 and three asterisks denote *p* < 0.001.

We used immunolabelling to examine the expression dynamics of DVL1-3 in proliferating human immortalised C25 myoblasts (Fig. 1A), early differentiating myocytes (Fig. 1B) and differentiated multinucleated myotubes (Fig. 1C). While immunolabelling was weak, we could discern that localisation of the DVL isoforms differed. During proliferation the location of DVL1 was both cytoplasmic and nuclear, while DVL2 was detected clearly in the nucleus and DVL3 was predominantly cytoplasmic (Fig. 1A). This localisation of DVL1-3 was retained through myogenic differentiation, with the notable exception that DVL2 appeared mainly cytoplasmic during the later stages of differentiation (Fig. 1B and C).

We also examined the expression profile of the *DVL* isoforms through myogenesis using RT-qPCR (Fig. 1D). mRNA levels of *DVL1*-*3* remained constant in proliferating C25 myoblasts and through myogenic differentiation, although there appeared to be a (non-significant) trend towards increased DVL1 and DVL3 expression as differentiation progressed. Successful myogenic differentiation was confirmed by a significant decrease in expression of a proliferation marker (*CD1*), but significant increases of early (*MYOGENIN*) and late (*MYHC*) differentiation markers (Fig. 1D).

### Knockdown of DVL1 or DVL3 significantly impairs human myoblast proliferation

**Figure 2:**
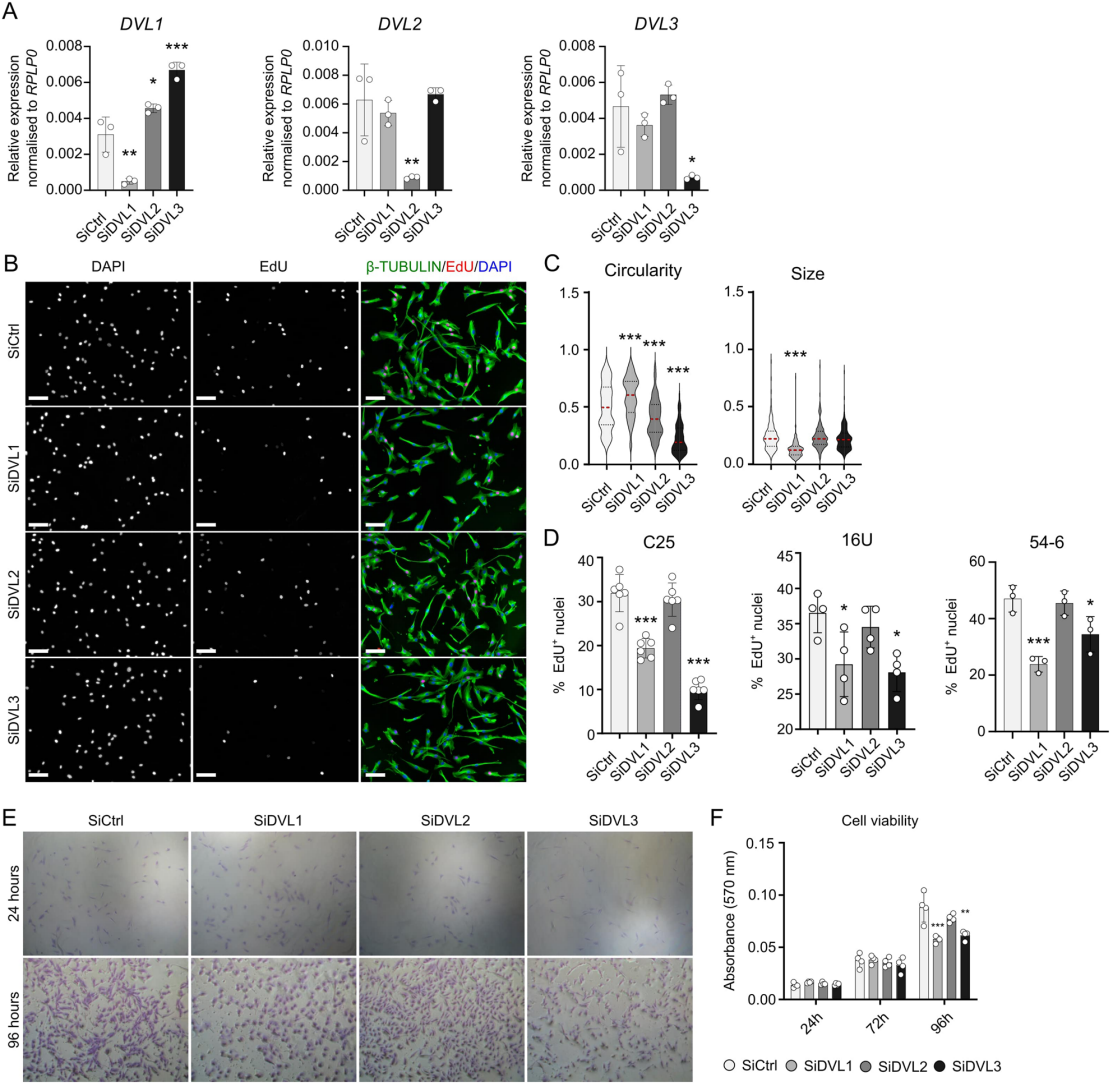
Knockdown of DVL1 or DVL3 reduces proliferation in human myoblasts. (**A**) Gene expression levels of *DVL1* (left), *DVL2* (middle) and *DVL3* (right) in proliferating C25 myoblasts after knockdown of each isoform for 48 h. *N* = 3 biological replicates. Data is represented as mean ± SD with significant differences calculated using a One-Way ANOVA with Dunett’s post-hoc test, comparing each group with the control (SiCtrl), where an asterisk denotes *p* < 0.05, two asterisks *p* < 0.01 and 3 asterisks *p* < 0.001. (**B**) Representative images of proliferating C25 myoblasts after knockdown of each DVL isoform for 48 h, immunolabelled for β-TUBULIN (green) to show cell morphology, with nuclei counterstained with DAPI (blue). Incorporated EdU is also visualised (red). Scale bar represents 100 µm. (**C**) Quantification cell circularity (0 equates to a straight line, 1 to a perfect circle) and size in proliferating C25 myoblasts after knockdown of each DVL isoform for 48 h, displayed as violin plots. Red line demarcates mean. Statistically significant differences were assessed using a One Way ANOVA with a Dunett’s post hoc test, comparing each knockdown individually to the control (SiCtrl). N = 135 cells from 3 independent experiments. Three asterisks denote *p* < 0.001. (**D**) Quantification of the percentage of human C25, 16U and 54–6 myoblasts that had incorporated EdU after knockdown of each DVL isoform. More than 200 nuclei were analysed for each of *N* = 3–6 wells. (**E**) Crystal violet staining of C25 myoblasts after DVL knockdown after 24 or 96 h of proliferation. (**F**) Quantification of crystal violet incorporation in C25 myoblasts after DVL knockdown after 24, 72 and 96 h of proliferation. *N* = 4. Data is represented as mean ± SD. Statistical differences were assessed using a One-Way ANOVA with Dunett’s post hoc test, comparing each knockdown group with the control, for each time-point, where two asterisks denote *p* < 0.01 and three asterisks *p* < 0.001.

Since all three *DVL* isoforms are expressed in proliferating human myoblasts, we performed siRNA-mediated knockdown of each *DVL* individually. RT-qPCR was performed to ensure that knockdown was specific to the target *DVL* isoform. As expected, a significant decrease in levels of the targeted *DVL* isoform occurred in each case for healthy control C25, 16U and 54–6 human myoblasts (Fig. 2A and Supplementary Fig. 1A). With exception of the targeted *DVL* isoform, no consistent changes in expression of the other *DVL* isoforms occurred in the three independent immortalised human myoblast lines. In the C25 cell line only, there was a potential increase in *DVL1* expression when *DVL2* was knocked down (Fig. 2A and Supplementary Fig. 1A) so we did not consider this a consistent effect.

Morphologically, C25 myoblasts appeared more rounded and smaller when *DVL1* was knocked down, while *DVL2* or *DVL3* knockdown caused a more elongated phenotype compared with control (Fig. 2B). Similar phenotypes were seen in 16U (Supplementary Fig. 1B) and 54-6 (Supplementary Fig. 1C) myoblasts after *DVL* knockdown. Quantification of cell circularity and size in proliferating C25 cells revealed that knockdown of *DVL1* resulted in a rounder myoblast morphology with a smaller area, while knockdown of either *DVL2* or *DVL3* caused a more elongated morphology, which was stronger with *DVL3* knockdown (Fig. 2C).

To investigate if knockdown of *DVL* isoforms affected proliferation, we performed a 2 h EdU pulse. Knockdown of *DVL1* or *DVL3* led to significantly less incorporation of EdU, so a reduced proliferation rate, while *DVL2* knockdown had no effect. This was a consistent observation across the three human myoblast lines C25, 16U and 54-6 (Fig. 2B, D and Supplementary Fig. 1B, C).

A crystal violet stain of C25 myoblasts after knockdown of each *DVL* isoform also demonstrated less cell density when *DVL1* or *DVL3* levels were reduced, compared to *DVL2* or controls (Fig. 2E). Absorbance of crystal violet as a proxy for cell viability was also measured in C25 myoblasts after knockdown of each *DVL* isoform at multiple time-points, and revealed a reduced amount of cells after knockdown of *DVL1* or *DVL3* for 96 h, compared to the control (Fig. 2F).

### DVL1 and DVL3 are required for myogenic differentiation

**Figure 3:**
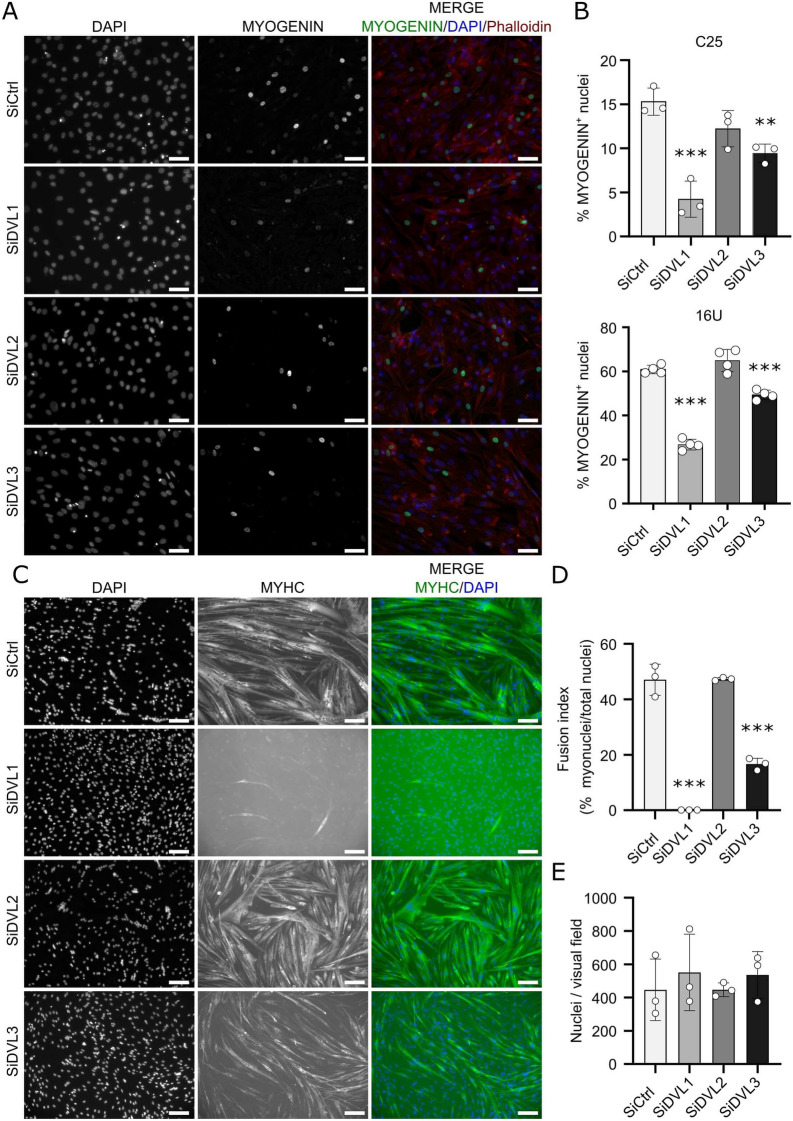
Knockdown of DVL1 or DVL3 retards myogenic differentiation in human myoblasts. (**A**) Differentiation was induced 48 h after DVL knockdown, and cells were fixed after 24 (C25) or 48 h of differentiation (16U). Representative images of differentiating C25 myocytes after knockdown of each DVL isoform, immunolabelled for MYOGENIN (green), with F-ACTIN (phalloidin, red) and nuclear counterstain DAPI (blue). Scale bars represents 50 µm. (**B**) Quantification of nuclei containing MYOGENIN in human C25 or 16U myocytes after knockdown of each DVL isoform. More than 200 nuclei were analysed for each *N* = 3–4 biological replicates. Data is represented as mean ± SD with significant differences calculated using a One-Way ANOVA with Dunett’s post-hoc test, comparing each group with the control (SiCtrl), where two asterisks indicate *p* < 0.01 and 3 asterisks *p* < 0.001. (**C**) Representative images of differentiating C25 myotubes after knockdown of each DVL isoform, immunolabelled for MYHC (green), with nuclei counterstained with DAPI (blue). Scale bar represents 100 µm. (**D**) Quantification of Fusion Index (myonuclei in myotubes with 2 or more nuclei/total nuclei) for C25 myotubes after knockdown of each DVL isoform, *N* = 3 biological replicates. (**E**) Number of nuclei per field in C25 myotubes after knockdown of each DVL isoform, *N* = 3 biological replicates. Data is represented as mean ± SD with significant differences calculated using a One-Way ANOVA with Dunett’s post-hoc test, comparing each group with the control (SiCtrl), where three asterisks indicate *p* < 0.001.

When myoblasts enter myogenic differentiation, they exit the cell cycle and so we investigated whether the decrease in the proliferation rate caused by knockdown of *DVL1* or *DVL3* was a consequence of precocious differentiation. MYOGENIN drives early myogenic differentiation and is expressed at high levels in myocytes as they prepare to fuse to form multinucleated myotubes. Knockdown of each *DVL* isoform was followed by immunolabelling for MYOGENIN. This revealed that early differentiating cultures (day 1 of differentiation in C25 and day 2 of differentiation in 16U) exhibited a marked reduction in the percentage of myocytes containing MYOGENIN after knockdown of *DVL1* or *DVL3*, while knockdown of *DVL2* had no effect (Fig. 3A and B, Supplementary Fig. 2). Thus knockdown of *DVL1* or *DVL3* not only significantly reduces the proliferation rate, but also interferes with entry into the myogenic differentiation program.

Terminal differentiation of C25 myoblasts was also reduced after knockdown of *DVL1* or *DVL3*, as determined through quantifying the fusion index (percentage of nuclei in MyHC-expressing myotubes with 2 or more nuclei). The phenotype was most pronounced after *DVL1* knockdown, when barely any multinucleated myotubes formed, but was also marked after knockdown of *DVL3* (Fig. 3C and D). To account for the reduced proliferation caused by *DVL1* or *DVL3* knockdown, cells were seeded at equal density before differentiation and quantification of nuclei per field confirmed that there was no significant difference in numbers (Fig. 3E). Thus, *DVL1* and *DVL3* are required for myogenic differentiation.

### Constitutive overexpression of DVL1 or DVL3 increases myoblast proliferation

**Figure 4:**
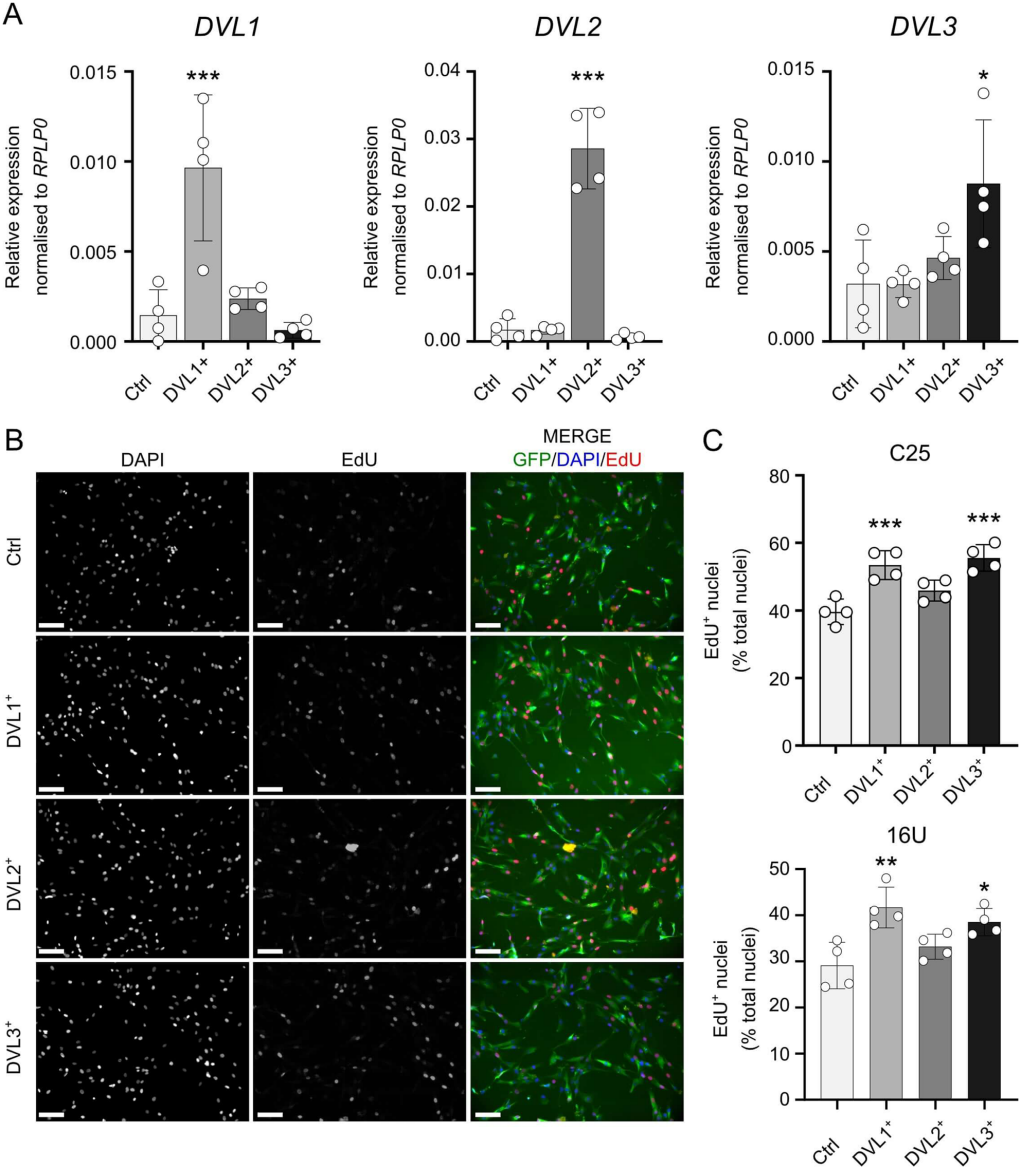
Constitutive overexpression of DVL1 or DVL3 enhances myoblast proliferation. (**A**) Gene expression levels of *DVL1* (left), *DVL2* (middle) and *DVL3* (right) in proliferating human C25 DVL1^+^, DVL2^+^ and DVL3^+^ myoblasts with stable lentiviral-mediated overexpression of each isoform. *N* = 4 biological replicates. Significant differences were calculated using a One-Way ANOVA with Dunett’s post-hoc test, comparing each group with the control (pUltra) where an asterisk denotes *p* < 0.05, two asterisks denote *p* < 0.01 and three asterisks is* p* < 0.001. (**B**) Representative images of proliferating C25 DVL1^+^, DVL2^+^ or DVL3^+^ myoblasts overexpressing the respective DVL isoform, immunolabeled for GFP from the lentiviral backbone (indicative of DVL levels) (green), with a DAPI nuclear counterstain (blue). Myoblasts with incorporated EdU (red) are also shown, after a 2 h EdU pulse performed 24 h after seeding. Scale bar represents 100 µm. (**C**) Quantification of C25 or 16U DVL1^+^, DVL2^+^ or DVL3^+^ myoblasts containing EdU as a percentage of total nuclei. More than 200 nuclei were analysed for each *N* = 4 biological replicate. Data is represented as mean ± SD with significant differences calculated using a One-Way ANOVA with Dunett’s post-hoc test, comparing each group with the control (pUltra), where one asterisk indicates *p* < 0.05, two asterisks indicate* p* < 0.01 and 3 asterisks* p* < 0.001.

As knockdown of *DVL1* or *DVL3* had such a drastic effect on proliferation, we wanted to understand if constitutive expression would have an opposite effect. We cloned each *DVL* isoform into a pUltra lentiviral backbone also encoding *eGFP* separated by a self-cleaving P2A site, so that the presence of GFP is correlated with that of the DVL isoform. We used these lentiviruses and FAC sorting to generate stably overexpressing human C25 myoblast lines termed DVL1^+^, DVL2^+^ or DVL3^+^. RT-qPCR confirmed that each *DVL* isoform was specifically overexpressed, and that overexpression of each *DVL* isoform did not affect expression of the other two (Fig. 4A).

We did not observe any overt morphological changes after constitutive overexpression of any *DVL* isoform in C25 (Fig. 4B) or 16U (Supplementary Fig. 3), in contrast to the effects of DVL1 or DVL3 knockdown (Fig. 2B and Supplementary Fig. 1B, C). We assessed the proliferation rate using a 2 h EdU pulse, as per the knockdown experiments. Constitutive *DVL1* or *DVL3* overexpression in DVL1^+^ or DVL3^+^ C25 or 16U myoblasts significantly increased the proliferation rate in both myoblast lines, while *DVL2* overexpression in DVL2^+^ myoblasts had no effect (Fig. 4B, C and Supplementary Fig. 3).

### DVL 1 or DVL3-mediated increase in proliferation is independent of WNT ligand stimulation

**Figure 5:**
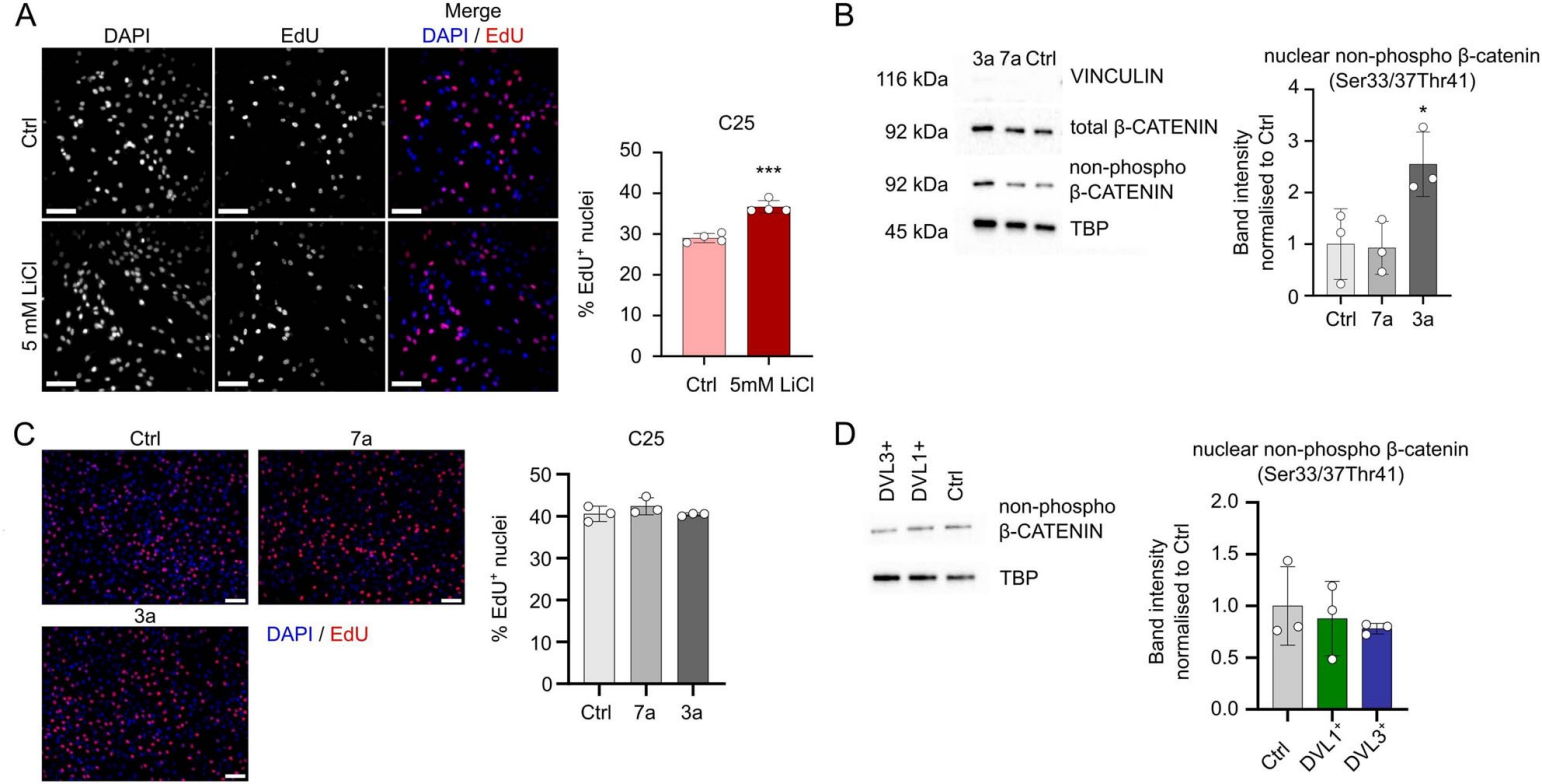
DVL-enhanced proliferation does not operate through β-CATENIN. (**A**) Representative images of proliferating C25 myoblasts after stimulation with 5 mM LiCl for 24 h showing incorporated EdU (red), with all nuclei counterstained with DAPI (blue). Scale bar represents 100 µm. Quantification was performed for 4 biological replicates, and significant difference was assessed using a student’s T-test. Three asterisks indicates *p* < 0.001. (**B**) Quantification of western blot for total β-CATENIN and non-phosphorylated active β-CATENIN in nuclear fractions of proliferating C25 myoblasts stimulated with WNT ligands WNT7a or WNT3a for 24 h. A representative blot is shown that includes cytoplasmic housekeeper VINCULIN, nuclear housekeeper TBP, total β-CATENIN and active β-CATENIN. Band intensity was normalised to the nuclear housekeeper TBP and changes are shown as fold change to unstimulated control. Labels indicate protein sizes. *N* = 3 independent biological replicates, significant difference between treatment and Ctrl was assessed using a One-Way ANOVA, with an asterisk denoting *p* < 0.05. (**C**) Representative images of proliferating C25 myoblasts stimulated with WNT ligands WNT7a or WNT3a for 24 h, with quantification of the percentage of EdU containing nuclei. *N* = 3, no significant differences were found using a One-Way ANOVA with Dunnett’s post-hoc test, comparing each group to the control. Scale bar equal 100 µm. (**D**) Western blot for non-phosphorylated active β-CATENIN in proliferating control C25, and C25 DVL1^+^ or DVL3^+^ myoblasts constitutively overexpressing DVL1 or DVL3. Band intensities were quantified and normalised to TBP, expression is shown as fold change to control (Ctrl). *N* = 3 independent biological replicates, no statistical differences observed between the overexpressing samples and the controls, assessed with a One-Way ANOVA with Dunnett’s post-hoc test, comparing each group to the control.

As DVL proteins are a core component of WNT signalling, we next tested if DVL-modulated effects on proliferation were a response to increased WNT signalling. LiCl inactivates GSK3β, allowing non-phosphorylated β-CATENIN to translocate to the nucleus and activate canonical WNT signalling^41^^–^^43^. We performed an EdU assay on proliferating C25 myoblasts that had been stimulated for 24 h with 5 mM LiCl prior to the 2 h EdU pulse. Activation of canonical WNT signalling significantly increased the proliferation rate of human C25 myoblasts, with the percentage of myoblasts incorporating EdU rising from 29 to 36.7% (Fig. 5A), as previously observed in murine satellite cells^44^ and immortalised C2C12 myoblasts^45^.

Since LiCl activates canonical WNT signalling downstream of DVLs, we next assessed if the same pro-proliferative effect occurred through stimulation with WNT ligands WNT7a or WNT3a. We first isolated protein from C25 myoblasts stimulated with 10 ng/ml WNT7a or WNT3a for 24 h and western blotted for total β-CATENIN and active non-phosphorylated (Ser33/37Thr41) β-CATENIN in the nuclear faction. Total β-CATENIN was unchanged between the WNT-stimulated and untreated controls. Phosphorylation of β-CATENIN catalyses degradation of the protein, and so only stabilised dephosphorylated β-CATENIN translocates into the nucleus. Stimulation with WNT3a increased levels of active non-phosphorylated β-CATENIN in the nuclear fraction (Fig. 5B, with the *N* = 3 full membranes shown in Supplementary Fig. 4). Given that LiCl stimulation increased proliferation, and WNT3a stimulation increased active nuclear β-CATENIN, it was unexpected that stimulation with WNT3a (or WNT7a) did not increase proliferation (Fig. 5C). Nuclear fractioning of C25 DVL1^+^ or DVL3^+^ myoblasts stably overexpressing *DVL1* or *DVL3* did not reveal an increase in active β-CATENIN, despite enhanced proliferation (Fig. 5D, with the *N* = 3 full membranes shown in Supplementary Fig. 5). Thus, increased proliferation via *DVL1* or *DVL3* appears governed through mechanisms other than facilitating translocation of β-CATENIN to the nucleus.

### DVL1 and DVL3 require the NLS signal to enhance proliferation in human myoblasts

**Figure 6:**
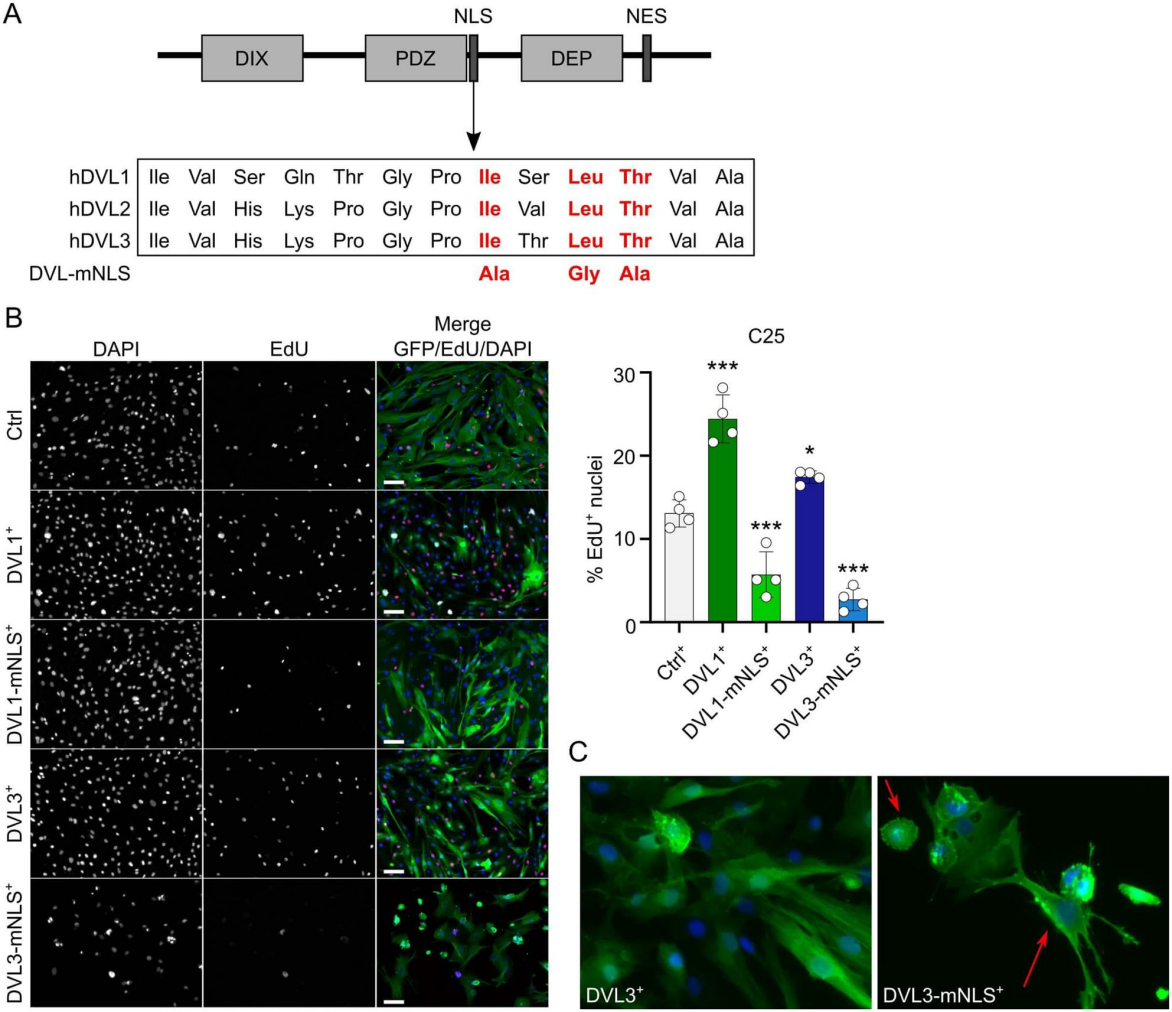
Mutation of the NLS sequence in DVL1 or DVL3 leads to reduced proliferation. (**A**) Schematic of the three conserved domains (DIX, PDZ, DEP) in DVL proteins, together with the positions of the nuclear localisation sequence (NLS) and the nuclear export sequence (NES), together with the changes to key amino acid residues to destroy the NLS (red text). (**B**) Representative images of proliferating C25 DVL1^+^, DVL1-mNLS^+^, DVL3^+^, DVL3-mNLS^+^ and control (Ctrl) myoblasts, with incorporated EdU (red), immunolabelled for GFP (green) from the lentivirus (correlated with DVL expression) and DAPI nuclei counterstain (blue). EdU pulse was performed 24 h after seeding stably overexpressing C25 myoblast lines. Scale bar represents 100 µm. Quantification was performed for 4 biological replicates, and significant difference was calculated using a One-way ANOVA with Dunnett’s post hoc test, comparing each group with the control, where an asterisk denotes *p* < 0.05 and 3 asterisks denotes *p* < 0.001. (**C**) Representative images of DVL3^+^ (left) or DVL3-mNLS^+^ (right) C25 myoblasts. Red arrows point to an unusually round, and an uncharacteristically elongated, DVL3-mNLS^+^ myoblast.

Even though levels of active non-phosphorylated β-CATENIN were not changed by overexpression of *DVL1* or *DVL3*, increased DVL expression may still enhance canonical WNT signalling in a less traditional way. DVL proteins in the nucleus form a complex with c-JUN and β-CATENIN on the promoter of WNT target genes, indicating that they can function as co-transcription factors^46^. Each DVL isoform contains a conserved nuclear localisation signal (Ile * Leu Thr) after the PDZ domain (Fig. 6A). We mutated this nuclear localisation signal to Ala * Gly Ala in *DVL1* and *DVL3* (since manipulation of *DVL2* expression had no effects) (Fig. 6A). The mutated *DVL* versions were stably overexpressed in C25 myoblasts to generate DVL1-mNLS^+^ and DVL3-mNLS^+^. Inactivating the NLS sequences caused DVL1-mNLS^+^ or DVL3-mNLS^+^ to act as dominant-negatives, causing proliferation to drop below control levels, similar to the effects observed after knockdown of endogenous *DVL1* or *DVL3* (Fig. 6B). Additionally, while C25 DVL1-mNLS^+^ myoblasts had generally normal morphology, overexpression of DVL3-mNLS had drastic effects: attachment of DVL3-mNLS^+^ myoblasts after seeding was compromised, and the cells that did attach were either small and round, or flattened with uncharacteristically long protrusions (Fig. 6B, C).

### DVL3 operates via the DIX and/or PDZ domain to enhance proliferation in human myoblasts

**Figure 7:**
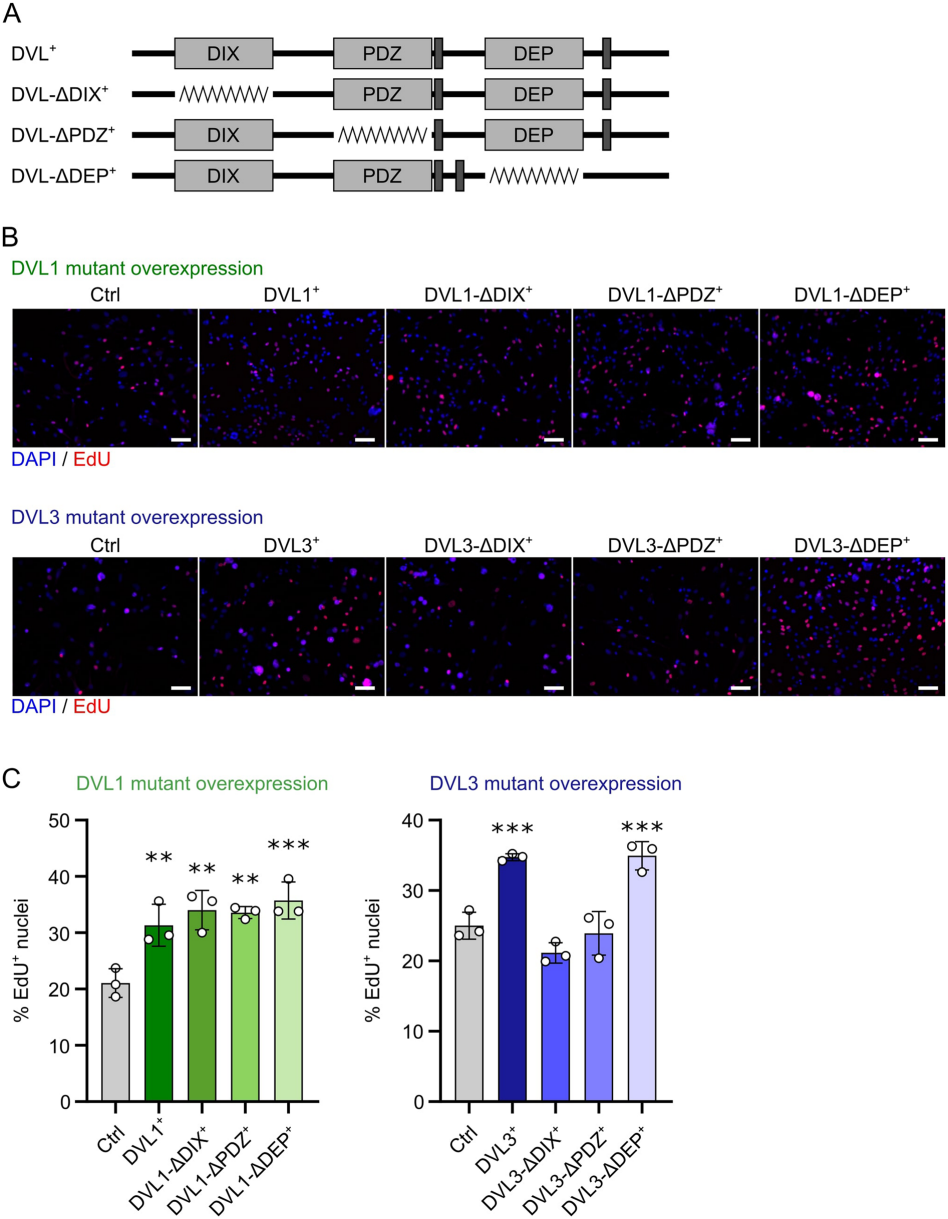
Deleting conserved domains in DVL1 or DVL3 has different effects on myoblast proliferation. (**A**) Schematic representation of the relative positions of the DIX, PDZ and DEP domains in full-length DVL protein and the respective domain deletion mutants. (**B**) Representative images of proliferating C25 myoblasts with stable overexpression (> 1 week) of wild type DVL1^+^ or DVL3^+^ and their respective mutants. An EdU pulse was performed 24 h after seeding stably overexpressing myoblast lines. Proliferating myoblasts with incorporated EdU (red), and all nuclei counterstained with DAPI (blue) are shown. Scale bar represents 100 µm. (**C**) Quantification of proliferation rates after overexpression of DVL1 or DVL3 and their respective mutants. A statistically significant difference was calculated using a One-Way ANOVA with Dunett’s post-test, comparing each sample to the control, where 2 asterisks denotes *p* < 0.01 and 3 asterisks denotes *p* < 0.001. Quantification was performed for 3 biological replicates.

In addition to their function at the cell membrane/in the cytoplasm of propagating canonical and non-canonical WNT signalling, the DIX and PDZ domains of DVL proteins are also implicated in the nuclear functions of DVL. DVL1 and DVL3 mutants were generated lacking each of the three conserved domains (DIX, PDZ and DEP) (Fig. 7A), and the effect of their overexpression on myoblast proliferation was assessed after lentiviral transduction and selection of stable myoblast lines. Specific deletion of each domain individually in DVL1-ΔDIX^+^, DVL1-ΔPDZ^+^ or DVL1-ΔDEP^+^ C25 myoblasts had no effect on the function of the protein in enhancing proliferation, as per overexpression of wildtype DVL1 in DVL1^+^ myoblasts (Fig. 7B, C). However, the pro-proliferative effect of *DVL3* overexpression in DVL3^+^ myoblasts relied on the DIX and/or PDZ domains, as removal of either in DVL3-ΔDIX^+^ or DVL3-ΔPDZ^+^ myoblasts caused the proliferation rate to return to control levels (Fig. 7B, C). Overexpression of *DVL3* lacking the DEP domain in DVL3-ΔDEP^+^ myoblasts increased their proliferation, as per overexpression of wild type DVL3 (Fig. 7B, C).

### Knockdown of DVL1 or DVL3 reduces proliferation in alveolar rhabdomyosarcoma cells

*DVL* expression is commonly increased in many types of cancer^41,47^^–^^49^. Rhabdomyosarcoma, a childhood onset cancer affecting soft tissue, shares significant similarities with skeletal muscle^50^. Considering the impact of manipulation of *DVL1* or *DVL3* on skeletal myoblast proliferation, we investigated the role of the DVL proteins in rhabdomyosarcoma. A published dataset investigating transcriptomic differences between five skeletal muscle and 101 rhabdomyosarcoma patient samples (GSE108022) found that VANGL2/RHOA, components of WNT signalling, were important in the regulation of growth and self-renewal of embryonal rhabdomyosarcoma (ERMS) cells^27^.

**Figure 8:**
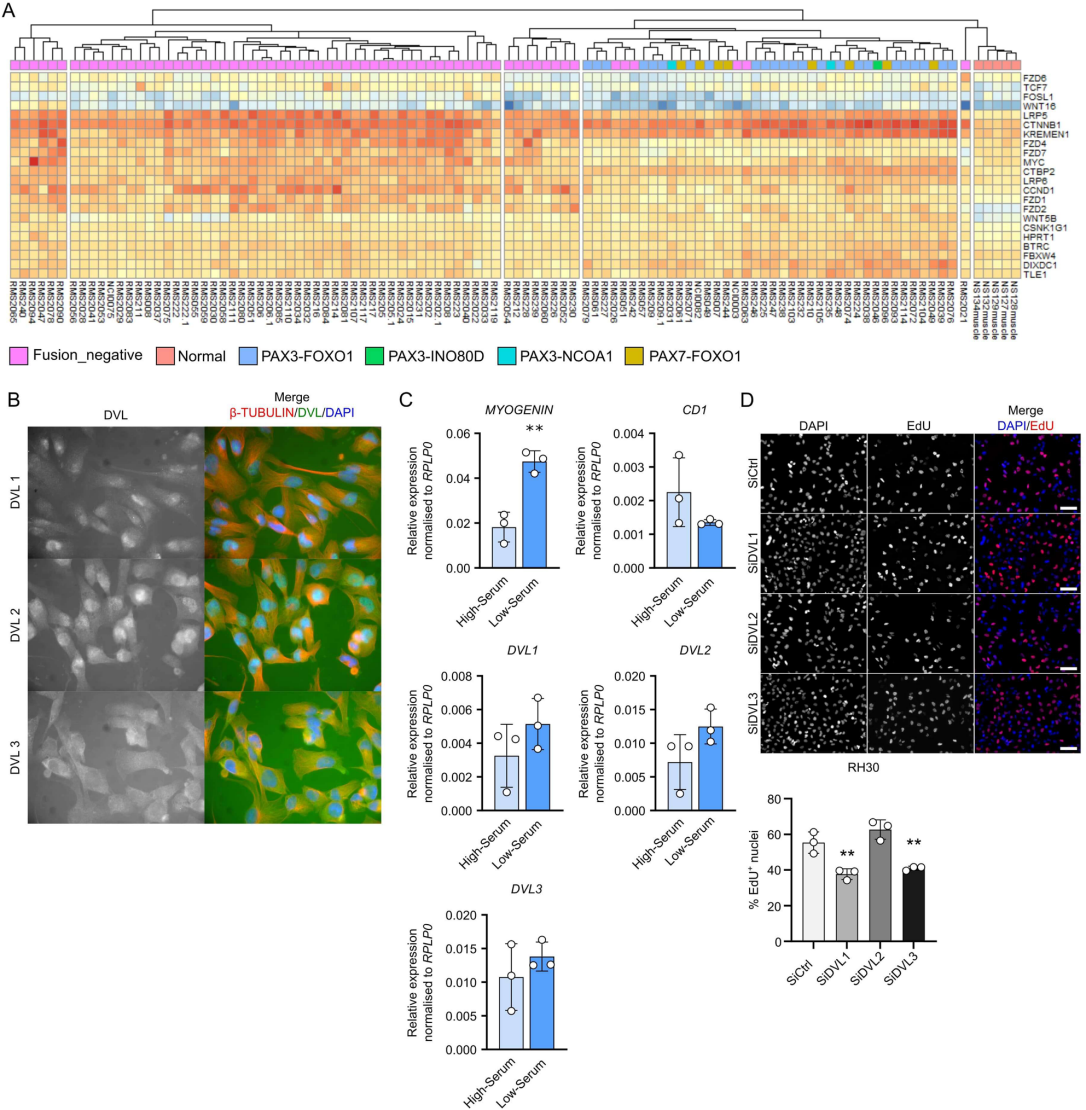
Knockdown of DVL1 or DVL3 reduces proliferation in alveolar rhabdomyosarcoma cells. (**A**) Heatmap of 22 differentially expressed genes associated with the WNT pathway in fusion-negative (pink) and fusion-positive (various colours) rhabdomyosarcoma biopsies and healthy muscle controls (Normal – orange) (GSE108022). (**B**) Localisation of DVL1-3 (green) in RH30 ARMS cells exposed to high-serum medium, co-immunolabelled for β-TUBULIN (red) with nuclei counterstained with DAPI (blue). (**C**) Gene expression profiles for *DVL1-3*, the proliferation marker *CD1* and the early differentiation marker *MYOGENIN* in RH30 cells exposed to high or low-serum medium. *N* = 3 independent biological replicates. Significant differences were assessed using a students unpaired two tailed T-test, comparing high-serum to low-serum culture conditions where two asterisks denote* p* < 0.01. (D) Representative images of proliferating RH30 cells that have incorporated EdU (red) after individual knockdown of each DVL isoform for 48 h, with nuclei counterstained with DAPI (blue). Scale bar represents 100 µm. Quantification of EdU containing cells as a percentage of total nuclei after knockdown of each DVL isoform. Statistically significant differences were calculated using a One-Way ANOVA with Dunett’s post-hoc test, comparing each group with the control (SiCtrl). A significant difference is denoted via two asterisks where *p* < 0.01. More than 200 nuclei were analysed for each of *N* = 3 biological replicates.

22 genes mapping to the WNT signalling pathway were differentially expressed between fusion negative ERMS, fusion positive ARMS and healthy skeletal muscle. Performing hierarchical clustering based on those 22 genes revealed six distinct clusters: ERMS samples were separated in 4 different clusters, all ARMS samples clustered together, mostly independent of their specific fusion state (PAX3-FOXO1, PAX3-NCOA1, PAX3-INO80D or PAX7-FOXO1), with the final group comprising the five healthy muscle samples (Fig. 8A). Since the fusion positive ARMS samples formed a single cluster, we further investigated the role of DVLs in RH30 cells, a PAX7-FOXO1 fusion positive ARMS cell line.

DVL protein was readily detectable using immunofluorescence in proliferating RH30 cells maintained in high serum medium (Fig. 8B). DVL1 and DVL2 were localised to the nucleus, while DVL3 appeared predominantly cytoplasmic (Fig. 8B). Low serum culture conditions induce differentiation in muscle cells, and similarly, RH30 cells increased *MYOGENIN* expression but *CD1* expression did not decrease significantly, showing that they remain proliferative (Fig. 8C). None of the three *DVL* isoforms showed any significant change in expression between high and low serum conditions (Fig. 8C).

After establishing that the DVL proteins were present in RH30 cells, we performed siRNA-mediated DVL knockdown and quantified the proliferation rate after a 2 h EdU pulse delivered 48 h later. Knockdown of *DVL1* or *DVL3* significantly reduced the proliferation rate of RH30 cells compared to siRNA control (SiCtrl), while knockdown of *DVL2* did not affect the percentage of cells that had incorporated EdU (Fig. 8D). This was the same effect that *DVL1* or *DVL3* knockdown had on the proliferation rate of human myoblasts (Fig. 2).

### DVL1 and DVL3 require nuclear localisation to enhance proliferation in alveolar rhabdomyosarcoma cells

**Figure 9:**
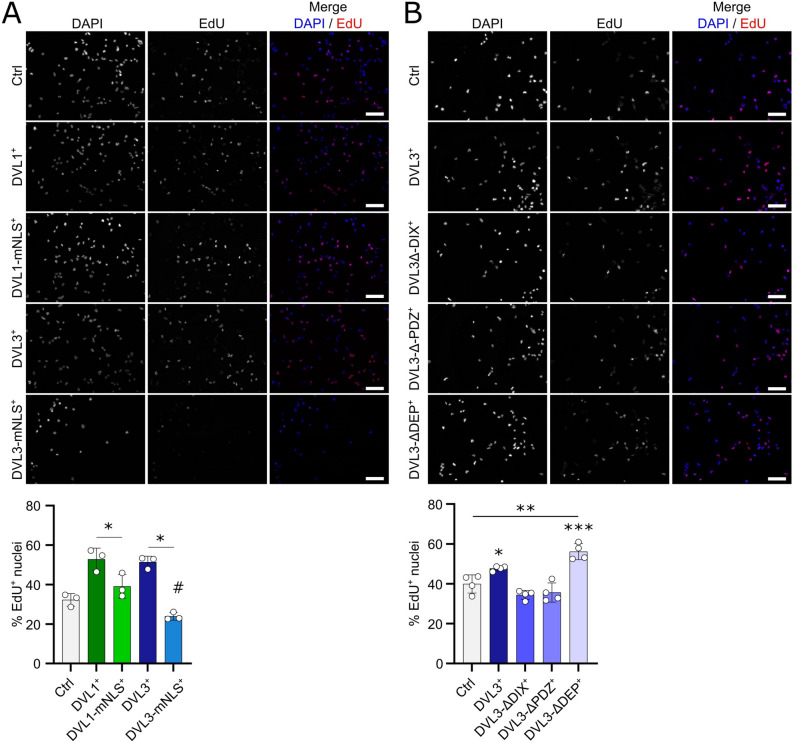
DVL1 and DVL3 require nuclear localisation to enhance proliferation in ARMS cells. (**A**) Representative images of stable proliferating DVL1^+^, DVL1-mNLS^+^, DVL3^+^, DVL3-mNLS^+^ and control (pUltra) RH30 ARMS cells with incorporated EdU (red) and a DAPI nuclear counterstained (blue). Scale bar represents 100 µm. Quantification was performed for 3 biological replicates, and a significant difference was calculated using a student’s T-test, where an asterisk denotes *p* < 0.05 comparing the two groups connected with a line and # denotes *p* < 0.05 compared to control. (**B**) Representative images of proliferating DVL3^+^, DVL3-ΔDIX^+^, DVL3-ΔPDZ^+^, DVL3-ΔDEP^+^ or control (pUltra) RH30 cells with EdU incorporation visualised (red) and a DAPI nuclear counterstained (blue). Scale bar represents 100 µm. Quantification was performed for 4 biological replicates, and significant difference calculated using a One-Way ANOVA, comparing each group against the control, where an asterisk denotes *p* < 0.05, and three asterisks denotes *p* < 0.001.

Even though RH30 cells are already highly proliferative, lentiviral-mediated stable overexpression of *DVL1* or *DVL3* further increased their proliferation rate, as shown by the higher percentage of cells that incorporated EdU after a 2 h pulse (Fig. 9A). To determine if the enhanced proliferation was dependent on the nuclear localisation of DVL1 or DVL3, we again used our *DVL1-mNLS* or *DVL3-mNLS* constructs*,* with the mutated NLS sequence. Constitutive overexpression in DVL1-mNLS^+^ or DVL3-mNLS^+^ RH30 cells prevented the increase in the proliferation rate observed when wild-type *DVL1* or *DVL3* were overexpressed (Fig. 9A). Interestingly, mutation of the NLS in DVL3-mNLS^+^ RH30 cells actually reduced the proliferation rate to below that of the control (Fig. 9A). Overexpression of the *DVL3-mNLS* also led to a reduced number of DVL3-mNLS^+^ RH30 cells compared to the other conditions (Fig. 9A). Since identical numbers of RH30 cells were seeded, this suggests that overexpression of *DVL3-mNLS* drastically reduced the proliferation rate, and/or prevented ARMS cells from attaching/caused cell death.

As the *DVL3-mNLS* mutant had a more pronounced effect compared with the *DVL1-mNLS* mutant, we stably overexpressed our *DVL3-*Δ*DIX*, *DVL3-*Δ*PDZ* and *DVL3-*Δ*DEP* mutants in RH30 cells. Deletion of the DIX or PDZ domain prevented *DVL3* from increasing the proliferation rate in DVL3-ΔDIX^+^ or DVL3-ΔPDZ^+^ RH30 cells (Fig. 9B). In contrast, deletion of the *DEP* domain in DVL3-ΔDEP^+^ RH30 cells actually enhanced proliferation, even over that achieved by overexpression of the full length DVL3 protein (Fig. 9B).

## Discussion

DVL proteins are predominantly known for their role as scaffolding proteins, acting between Frizzled receptors and the β-CATENIN destruction complex in WNT signalling. During active WNT signalling, DVL proteins are phosphorylated to inhibit the destruction complex, allowing non-phosphorylated β-CATENIN to translocate to the nucleus^11,12^. However, a dual role for DVL at both the cell membrane/in cytoplasm and nucleus has been established^52^. The presence of DVL in the nucleus is necessary for β-CATENIN-dependent downstream gene expression^15^, including through activation of TCF/LEF-mediated transcription together with c-JUN.

In this study, we show that DVL1 and DVL3, but not DVL2, regulate proliferation in human myoblasts and are necessary for successful myogenic differentiation. Indeed, DVLs are requisite for efficient myoblast function as when either DVL1 or DVL3 are knocked down, both the proliferation rate, entry into myogenic differentiation and fusion are compromised. We also report that DVLs are required for maximal proliferation in ARMS cells: consistent with observations that DVL overexpression is associated with higher proliferation, invasiveness and aggressiveness in breast cancer^47,53^, prostate cancer^54^, primary cervical squamous cancer^55^ and high-grade gliomas^56^. Curiously though, it is the actions of DVL1 or DVL3 in the nucleus that facilitates these effects on proliferation in myoblasts and ARMS.

DVL proteins exhibit head to tail dimerisation via the DIX domain and form large protein puncta 1–2 µM in size when overexpressed^57,58^. That puncta assemble, strongly suggests that DVL proteins can dimerise. Additionally, upon WNT stimulation, DVL2 concentration at the plasma membrane increases and the protein oligomerises^28^. We found that mutating the NLS sequence reduced the cell proliferation rate, showing that nuclear localisation is important. This may be a direct effect of the mutant NLS on DVL1 or DVL3, and/or the mutant DVLs may dimerise/oligomerise with endogenous wildtype DVL, preventing it from operating. This would explain why there is a reduction in the proliferation rate, below even the control level.

Interestingly, we did not observe an increase in nuclear β-CATENIN in response to DVL overexpression, suggesting that the pro-proliferative effects of DVL1 or DVL3 are not only due to nuclear translocation of β-CATENIN. However, nuclear β-CATENIN is present in unstimulated cells, and so the pool of nuclear β-CATENIN may already be sufficient to drive higher levels of WNT signalling when levels of co-activators such as DVL increase. Since DVLs facilitate β-CATENIN dependent transcription via TCF/LEF transcription factors within the nucleus^15,16,52^, overexpressing DVLs could further increase the transcriptional output of canonical WNT signalling. ChIP-Seq to further understand the putative role of DVLs as transcription factor/co-factors would be informative.

A consistent finding was that manipulating either DVL1 or DVL3 had similar effects, while manipulation of DVL2 did not measurably affect myoblast behaviour. Similarly, knockdown of DVL1 or DVL3 in non-muscle cell lines HEK293T (human embryonic kidney cells) or P19 cells (embryonic carcinoma cell line) has a stronger effect on canonical WNT signalling than DVL2 knockdown^26^. The DIX and PDZ domains of DVLs are involved in propagation of canonical WNT signalling (reviewed in^33^), which would explain why their deletion abrogated the effect of DVL3 overexpression. Deletion of individual domains does not inhibit nuclear localisation of DVLs however^15^, so while DVL3 lacking the DIX or PDZ domain may still localise to the nucleus, they are then incapable of functioning effectively in either myoblasts or ARMS cells. However, the DEP domain is necessary for membrane recruitment of DVL^59^, which likely explains why deletion of the DEP domain did not alter DVL3 function. Therefore, DVL3 regulates myoblast proliferation via its nuclear localisation and likely ability to then propagate canonical WNT signalling via its DIX or PDZ domains.

In contrast to DVL3, provided that DVL1 is able to enter the nucleus, mutations of either the DIX, PDZ or DEP domains have no effects on its ability to enhance the proliferation rate in myoblasts. Thus, while manipulation of DVL3 or DVL1 led to the same biological outcome in myoblasts, they employ different mechanism. Two acetylated lysine residues in the PDZ and DIX domain of DVL1 also promote nuclear localisation (in addition to the NLS) and promoter binding^60^. If those residues can compensate for each other, then deletion of both amino acids/domains would be required to inhibit the pro-proliferative effect of DVL1 overexpression. In DVL3, lysine residues do not occupy these two positions, which may explain the different mechanism of action.

Manipulation of DVL2 had no effects on proliferation in myogenic or ARMS cells. DVL2 is the most abundantly expressed DVL isoforms in multiple cell lines, such as murine F9 and P19 cells and human HEK293 cells^26^. Given that siRNA-mediated knockdown had a maximum efficiency of 85%, it is possible that the remaining pool of DVL2 was still sufficient to maintain its function. In murine skeletal muscle^61^ and immortalised muscle C2C12 cells^24,27^, protection of DVL2 from autophagy increases canonical WNT signalling which in turn increases differentiation. Those studies show significant effects during muscle regeneration and differentiation, but since our experiments were predominantly restricted to proliferation, it is possible that the effect of DVL2 manipulation would only become evident in mature muscle, and this late stage of myogenesis cannot be reliably modelled in our 2D cell culture system.

Co-immunoprecipitation to identify DVL interactors in the nucleus would further pinpoint mechanistic differences between DVL1 and DVL3. Additionally, considering the importance of the PDZ and DIX motifs for proliferation in ARMS cells, specific inhibitors could be tested as part of potential therapies for this difficult to treat sarcoma.

## Conclusions

We demonstrate the role of DVL1 and DVL3 in proliferation of human myoblasts and ARMS cells. Knockdown of either DVL1 or DVL3 significantly reduced the proliferation rate, while constitutive expression of either DVL1 or DVL3 enhanced proliferation. DVL1 or DVL3 knockdown also inhibited myoblasts from entering myogenic differentiation and forming multinucleated myotubes. This process was independent of WNT ligand stimulation, as addition of either WNT3a or WNT7a did not change the cell proliferation rate. A functional nuclear localisation signal was essential to maintain the pro-proliferative effect of either DVL isoforms, however the mechanism of how DVL1 or DVL3 overexpression increases proliferation diverges between the two isoforms. Removal of the DIX, PDZ or DEP domain from DVL1 did not interfere with its capacity to increase proliferation, while removal of the DIX or PDZ domain from DVL3 abrogated its ability to enhance cell proliferation.

## Materials and methods

### Cell lines

Immortalised human myoblast lines C25 and 54–6 were obtained from the Institute de Myologie (France)^62,63^, while the 16U line was from the UMMS Wellstone Centre for FSHD (USA). The RH30 ARMS cell line (CVCL_0041) was from the Institute of Cancer Research, London^64^. All cell lines were regularly tested for mycoplasma, and each batch were cultured for less than 3 weeks.

### Cell culture

C25, 16U, 54-6 cells were cultured in Promocell skeletal muscle growth medium (Promocell, C-23060) supplemented with 15% foetal calf serum (FBS) and 1:1000 Gentamycin (Sigma). Differentiation was induced by switching the medium to DMEM GlutaMax, 0.5% FBS, 1:1000 bovine Insulin (Sigma) and 1:1000 Gentamycin. RH30 were cultured in DMEM GlutaMax (Gibco, 10566016) supplemented with 10% FBS and 1% Pen/Strep (Sigma). Cells were maintained in a humidified incubator at 37 °C and 5% CO_2_. Differentiation medium was used for RH30 cell culture under low serum conditions.

For WNT ligand stimulation experiments, cells were plated in 6 well plates and 24 h later were stimulated with 10 ng/µl WNT3a or WNT7a (final concentration) and protein harvested 24 h later.

### Generation of lentiviral plasmids and overexpression

The mRNA sequence for *DVL1* (NM_031820), *DVL2* (NM_004422) and *DVL3* (NM_007889) were obtained from NCBI gene (https://www.ncbi.nlm.nih.gov/gene) and used to design primers including restriction enzyme sites (Table 1). DVLs were then amplified from C25 myoblast mRNA, with XbaI and BamHI used for cloning *DVL1* and *DVL3*, and XbaI and EcoRI for *DVL2*. Purified and restriction enzyme-digested transcript was ligated into the lentiviral pUltra backbone obtained from Addgene. pUltra was a gift from Malcolm Moore (Addgene plasmid # 24129)^65^. Plasmids containing *DVL1* and *DVL3* with a mutated NLS sequence were custom designed and obtained from VectorBuilder.

**Table 1:** Primer sequences for cloning DVLs into the pUltra backbone.

*DVL1* Fwd	5’-ATA TCT AGA ATG GCG GAG ACC AAG ATT ATC T-3’
*DVL1* Rev	5’-ATA GGA TCC TCA CAT GAT GTC CAC GAA GAA CTC-3’
*DVL2* Fwd	5’-ATA TCT AGA ATG GCG GGT AGC AGC ACT-3’
*DVL2* Rev	5’-ATA GAA TTC CTA CAT AAC ATC CAC AAA GAA CTC GCT GGG-3’
*DVL3* Fwd	5’-ATA TCT AGA ATG GGC GAG ACC AAG ATC ATC TAC C-3’
*DVL3* Rev	5’-ATA GGA TCC TCA CAT CAC ATC CAC AAA GAA CTC ACT GG-3’

Viral particles were generated using HEK293T cells transfected with pUltra and packaging plasmids: pRSV-REV (Addgene, #12253), PMD2.G (Addgene, #12259) and pMDLg/pRRE (Addgene, #12251).

For overexpression, 20 000 cells were transduced with 0.5 ml supernatant containing viral particles. GFP expression was observed 24–48 h later and cell lines were passaged at least 2 times before being plated for experiments. Transduction was performed in myogenic proliferation medium except for RH30 cells, for which DMEM based proliferation medium was used.

### Immunolabelling

Cells were fixed with 4% paraformaldehyde (PFA)/PBS for 10 min, permeabilized with 0.05% Triton/PBS for 10 min and non-specific antibody binding blocked with 10% goat serum/PBS for 30 min. Cells were then incubated with the primary antibody in PBS/1% goat serum (Table 2) at 4 °C overnight followed by three PBS washes and incubation with the secondary antibody (Invitrogen, 1:500) for 1 h at room temperature. After further PBS washes, nuclei were stained with DAPI (1:1000) and mounted for viewing and imaging on an aging Zeiss AxioVert 200 M epifluorescence microscope with a Zeiss AxioCam HRm and AxioVision 4.4 software (Zeiss, Jena, Germany).

**Table 2:** Primary antibodies used for immunolabelling.

Antibody	Reference/source	Working dilution
Polyclonal rabbit anti-DVL1	ab233003, Abcam	1:400
Polyclonal rabbit anti-DVL2	ab228804, Abcam	1:400
Monoclonal rabbit anti-DVL3	ab76081, Abcam	1:400
Monoclonal mouse anti-MYOGENIN	F5D, DSHB	1:10 (Supernatant)
Monoclonal mouse anti-β-TUBULIN	E7, DSHB	1:500
Monoclonal mouse anti-MyHC	MF20, DSHB	1:500
Polyclonal chicken anti-GFP	Abcam, ab13970	1:2000

For proliferation rate analysis, cells were pulsed with EdU for two hours prior to fixation. EdU incorporation was visualised using the Click-iT EdU Imaging Kit (Life Technologies) as per manufacturer’s instructions. EdU positive nuclei were counted and expressed as the percentage of total nuclei within the field.

### Cell size and circularity calculation

Proliferating cells were fixed and immunolabelled for β-TUBULIN. Shape and circularity were calculated using Fiji^66^ by drawing around individual cells and measuring the parameters. Circularity is represented as a value ranging from 0 to 1, where 0 is a straight line and 1 is a perfect circle.

### SiRNA mediated knockdown

80 000 cells were transfected with 1.5 nM siRNA (final concentration) against *DVL1* (Qiagen, SI02633183, mix of 4 different siRNA), *DVL2* (Qiagen, SI00063448, mix of 4 different siRNA), *DVL3* (Qiagen, SI00063476, mix of 4 different siRNA) or scrambled control siRNA for 24 h. 1.5 nM siRNA were incubated with 150 µl OptiMem (ThermoFisher Scientific, 31985062) and 9 µl Lipofectamine RNAiMax (ThermoFisher Scientific, 13778075) with 150 µl OptiMem. The solutions were combined, incubated for 20 min at room temperature and 250 µl were added to each well, already containing 1.75 ml fresh growth medium. 24 h after transfection, cells were washed, fixed and immunolabelled.

### Crystal violet staining

50 000 cells were seeded in 6 wells and allowed to adhere overnight. 24, 72 and 96 h after seeding, cells were washed with PBS, fixed with 4% PFA/PBS for 15 min, washed with double distilled (dd)H_2_O and stained with 0.1% crystal violet (50 mg crystal violet (B21932-14, Alfa Aesar) dissolved in 5 ml ethanol/45 ml ddH_2_O) for 20 min. Cells were then washed thrice with ddH_2_O, ddH_2_O was then aspirated and cells allowed to air dry. 2 ml 10% acetic acid was added to each well and the plate was incubated on a shaker for 20 min. The supernatant was diluted 1:5 and measured at 570 nm.

### RNA extraction and RT-qPCR analysis

mRNA was isolated from proliferating myoblasts and RH30 cells using the RNeasy kit (Qiagen, 74,104) according to manufacturer’s instructions. mRNA was reverse transcribed using the Quantitect reverse transcription kit (Qiagen, 205311) and SYBR green qPCR was performed (Takyon, UF-NSMT-B101), using the Viia7 machine (ThermoFisher). Relative gene expression was normalized to housekeeper *RPLP0*, and results are shown as 2^-ΔCT^. Primer sequences are: *RPLP0*, 5′-TGGTCATCCAGCAGGTGTTCGA-3′ (forward) and 5′-ACAGACACTGGCAACATTGCGG-3′ (reverse); *MYOGENIN*, 5′-CCAGGGGTGCCCAGCGAATG-3′ (forward) and 5′-AGCCGTGAGCAGATGATCC-3′ (reverse); *MyHC*, 5′-AGCAGGAGGAGTACAAGAAG-3′ (forward) and 5′-CTTTGACCACCTTGGGCTTC-3′ (reverse) The *MyHC* primers will amplify *MYH3*, *MYH8* and *MYH2*; *CD1*, 5′-GCTGTGCATCTACACCGACA-3′ (forward) and 5′-TTGAGCTTGTTCACCAGGAG-3′ (reverse); *DVL1*, 5’-CACCTCATCCAGACTCATCC-3’ (forward) and 5’-TCAAAGTTCACGTCATTCACC-3’ (reverse); *DVL2*, 5’-CACCTTTACCTCCTTTGCC-3’ (forward) and 5’-ATGCTCACTGCTGTCTCTC-3’ (reverse); *DVL3*, 5’-GCGAGACCAAGATCATCTACC-3’ (forward) and 5’-ATCTCCTCCTTCACCACTCC-3’ (reverse).

### Protein extraction and western blot

Protein and subcellular fractioning were performed according to the ABCAM protocol. In brief: plated cells were washed with ice cold PBS, scraped, lysed in fractioning buffer (20 mM HEPES, 10 mM KCl, 2 mM MgCl_2_, 1 mM EDTA, 1 mM EGTA and freshly added 1 mM DTT and PI Cocktail III 1:200) and incubated on ice for 15 min. Samples were passed through a 27G needle until cells were lysed and incubated on ice for 20 min. Samples were centrifuged at 720 g for 5 min and the supernatant containing the cytoplasm fraction collected. The nuclear pellet was washed in 500 µl fractioning buffer, passed through a 23G needle 10 times and centrifuged for 10 min at 720 g. The supernatant (containing the nuclear proteins) was collected. Supernatants containing nuclear or cytoplasmic proteins were mixed with 4 × Laemmli buffer, boiled for 5 min at 95 °C and frozen at − 20 °C for storage. Protein and 5 µl precision plus protein standards dual colour ladder (BioRad, #161–0374) were loaded on a 4–20% precast gel (BioRad, #4561094), run for 1 h at 60 V and transferred to nitrocellulose membrane at 70 V for 1 h. The membrane was cut to immunolabel for β-CATENIN and controls VINCULIN (Cell Signaling, #13901) or TBP (Cell Signaling, #8515). The strip containing β-CATENIN was first probed for active non-phospho β-CATENIN (Cell Signaling, #8814S), then stripped (ThermoFisher, 21059) and re-probed for total β-CATENIN (Cell Signaling, #L87A12). VINCULIN was used as housekeeper for the cytoplasmic fraction and TBP as housekeeper for the nuclear fraction. Band intensities were quantified and normalised to the housekeeper intensity.

### RNA-seq data analysis

Hayes et al.^51^ generated an RNA-seq dataset (GSE108022) analysing 106 samples: 5 normal muscle and 101 fusion-positive and fusion-negative RMS samples. DESeq analysis identified 22 genes attributed to the WNT signalling pathway (GSEA WNT_SIGNALLING) that were differently regulated between healthy muscle and RMS samples. The heatmap of those genes was generated using the pheatmap package in R.

## Supplementary Information

Supplementary Information.

## Abbreviations

ARMS: Alveolar rhabdomyosarcoma
DVL: Dishevelled
ERMS: Embryonal rhabdomyosarcoma
FDZ: Frizzled
NLS: Nuclear localisation signal
NES: Nuclear export signal
PCP: Planar cell polarity
TCF/LEF: T-cell factor/lymphoid enhancer factor
