# Supplementary material for: DVL1 and DVL3 require nuclear localisation to regulate proliferation in human myoblasts
Source: Sci Rep. 2022 May 19;12:8388. doi: 10.1038/s41598-022-10536-x (PMC9120025; doi:10.1038/s41598-022-10536-x)
Supplement: Supplementary file 1 — Supplementary Information. [file 41598_2022_10536_MOESM1_ESM.pdf]

DVL1/3 require nuclear localisation to regulate proliferation in human myoblasts

Johanna Pruller, Nicolas Figeac and Peter S. Zammit *

King's College London, Randall Centre for Cell and Molecular Biophysics, London, SE1 1UL, UK,

***** Corresponding author: peter.zammit@kcl.ac.uk

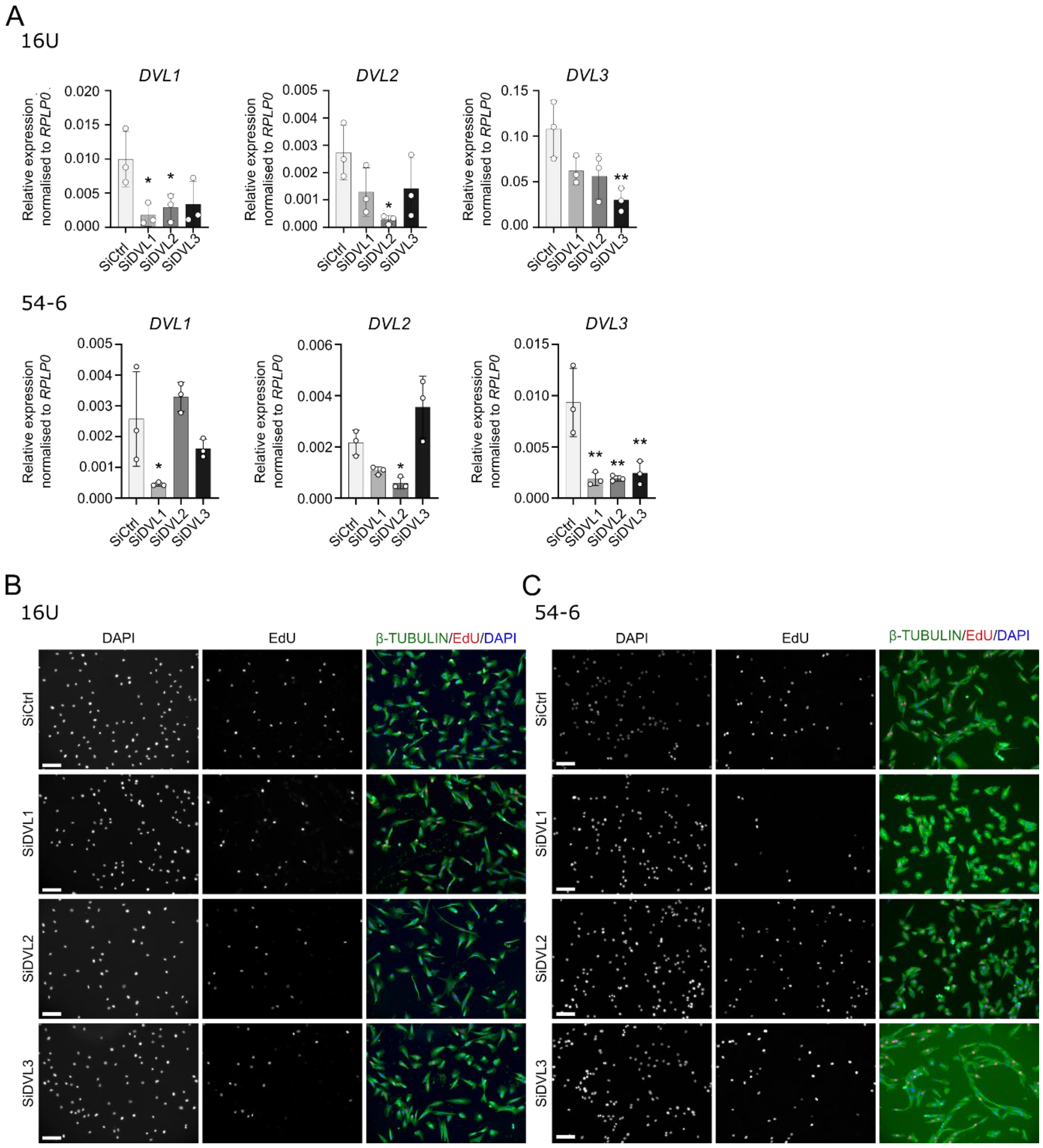

Supplementary Figure 1: (A) Gene expression levels of *DVL1* (left), *DVL2* (middle) and *DVL3* (right) in proliferating 16U (top) and 54-6 (bottom) myoblasts after knockdown of each isoform. N = 3 biological replicates. Data is represented as mean ± SD with significant differences calculated using a One-Way ANOVA with Dunett's post-hoc test, comparing each group with the control (SiCtrl), where an asterisk denotes $p < 0.05$, two asterisks $p < 0.01$ and 3 asterisks $p < 0.001$. (B,C) Representative images of proliferating 16U (B) and 54-6 (C) myoblasts after knockdown of each DVL isoform, immunolabelled for β-TUBULIN (green) to show cell morphology and nuclei counterstained with DAPI (blue). Myoblast with incorporated EdU are shown (red). Scale bar represents 100 µM.

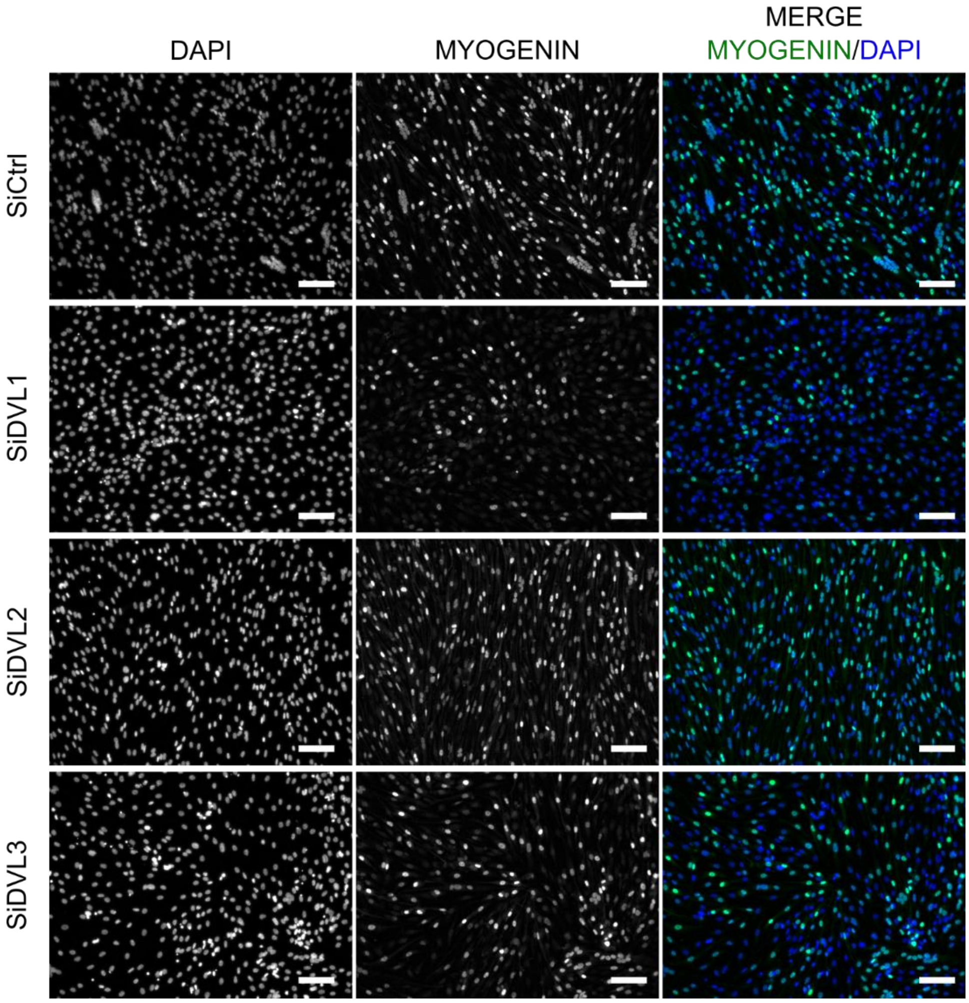

Supplementary Figure 2: Representative images of differentiating 16U myocytes after knockdown of each *DVL* isoform, immunolabelled for MYOGENIN (green), and nuclei counterstained with DAPI (blue). Scale bar represents 100 µM.

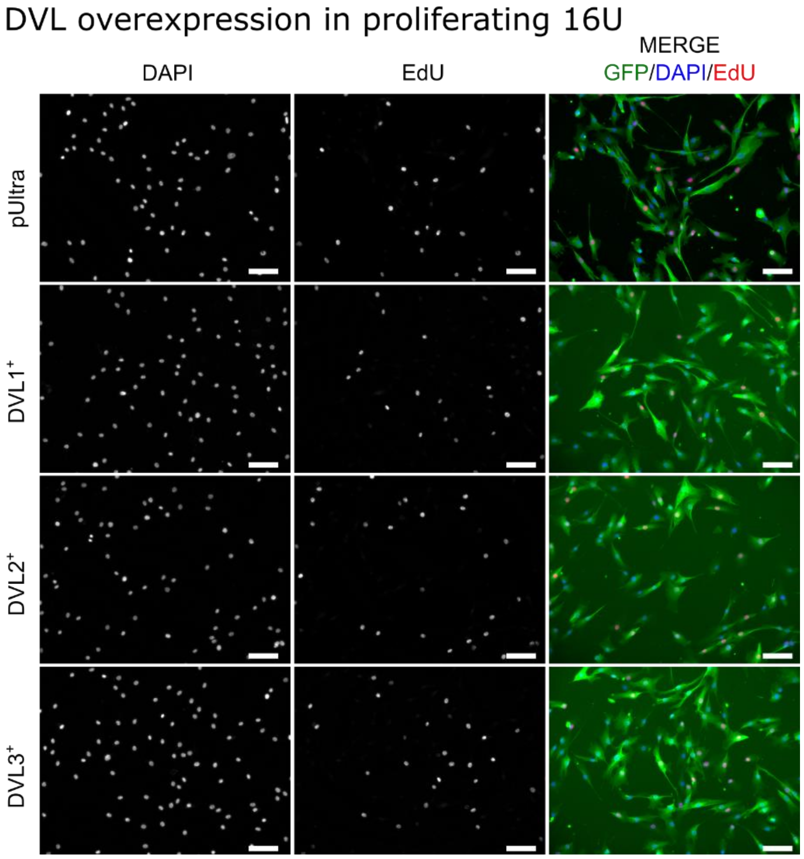

Supplementary Figure 3: Representative images of proliferating 16U after overexpression of each DVL isoform, immunolabelled for β-TUBULIN (green) to show cell morphology and nuclei counterstained with DAPI (blue). Myoblasts with incorporated EdU are shown (red). Scale bar represents 100 µM.

A

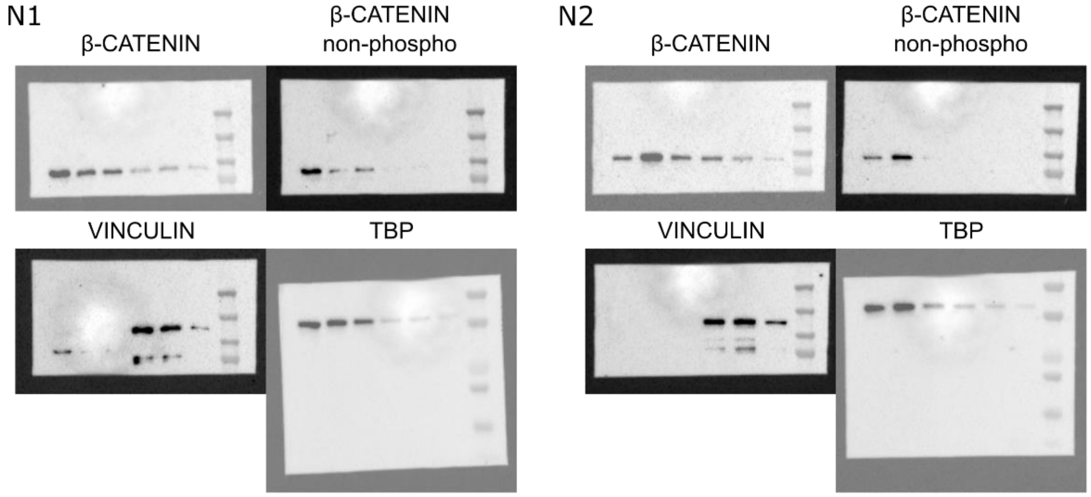

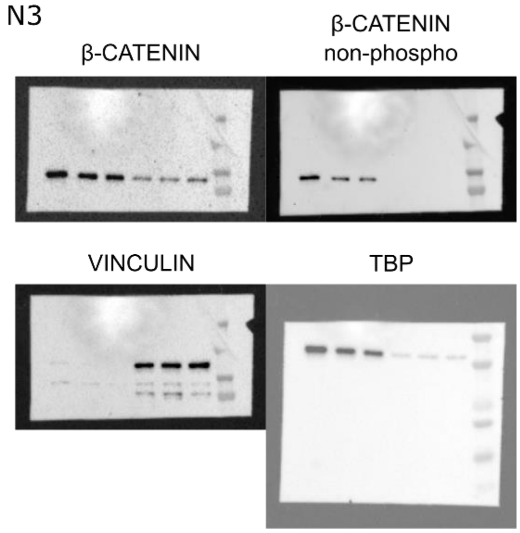

Order of samples:
(left to right)

**N1/N3:**	**N2:**
NF +Wnt3a	NF + Wnt7a
NF +Wnt7a	NF + Wnt3a
NF Ctrl	NF Ctrl
CF + Wnt3a	CF + Wnt3a
CF + Wnt7a	CF + Wnt7a
CF Ctrl	CF Ctrl

CF = cytoplasmic faction, NF = nuclear faction

B

	**β-CATENIN non-phospho**			**TBP**		
	**N1**	**N2**	**N3**	**N1**	**N2**	**N3**
**Ctrl**	2913.54	271.21	3411.55	6427.33	3184.13	5958.84
**+ Wnt3a**	12686.02	9563.59	9153.05	10164.6	10160.4	7361.08
**+ Wnt7a**	1740.72	3180.67	4028.55	10483.9	12216.7	10885.2

β-CATENIN/TBP fold change to Ctrl

**Ctrl**	1.22	0.23	1.55
**+ Wnt3a**	0.46	0.84	1.477
**+ Wnt7a**	3.26	2.11	2.27

Supplementary Figure 4: (A) Full membranes for Western blots (N1 - 3) shown in Figure 5B. Membranes were cut, the top part sequentially stained for non-phospho β-CATENIN, then β-CATENIN and then VINCULIN. The membrane was stripped between stainings for active and in-active β-CATENIN. The bottom part was stained against TBP. Samples stimulated with Wnt3a and Wnt7a are loaded in a different order in N2. (B) Densitometry measurements for non-phosphorylated β-CATENIN and TBP bands.

A

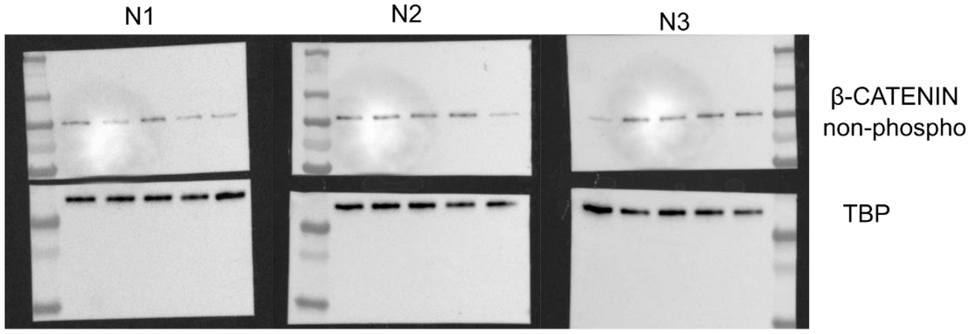

Order of samples:
(left to right)

pUltra
DVL1$^+$
DVL3$^+$
DVL1-mNLS$^+$
DVL3-mNLS$^+$
CF = cytoplasmic faction, NF = nuclear faction

B

	**β-CATENIN non-phospho**			**TBP**		
	**N1**	**N2**	**N3**	**N1**	**N2**	**N3**
**Ctrl**	777.57	1603.94	1407.82	3987.79	7642.23	3825.62
**DVL1$^+$**	656.38	2138.67	1613.31	5293.32	6247.52	5290.86
**DVL3$^+$**	1032.11	1718.255	1255.94	5573.03	8192.23	6130.15

**β-CATENIN/TBP fold change to Ctrl**			
**Ctrl**	0.76	0.79	1.43
**DVL1$^+$**	0.64	1.2	0.83
**DVL3$^+$**	0.95	1.03	0.56

Supplementary Figure 5: (A) Full membranes for Westernblots (N1 – 3) shown in Figure 5D. Membranes were cut, the top part stained for non-phospho β-CATENIN, and bottom part for TBP. (B) Densitometry measurements for non-phosphorylated β-CATENIN and TBP bands.
